# Hairy Roots of *Scutellaria* spp. (*Lamiaceae*) as Promising Producers of Antiviral Flavones

**DOI:** 10.3390/molecules26133927

**Published:** 2021-06-27

**Authors:** Anna Yurievna Stepanova, Aleksandra Ivanovna Solov’eva, Maria Victorovna Malunova, Svetlana Andreevna Salamaikina, Yury Mikhailovich Panov, Andrey Aleksandrovich Lelishentsev

**Affiliations:** 1 Group of Specialized Root Metabolism, K.A. Timiryazev Institute of Plant Physiology RAS, 127276 Moscow, Russia; slvjova.aleksandra@rambler.ru (A.I.S.); mmalunova@yandex.ru (M.V.M.); svetlana.salamaikina@gmail.com (S.A.S.); 2 Faculty of Chemistry, Moscow State University, 119991 Moscow, Russia; juriyypanov@rambler.ru; 3 Bioanalytical Laboratory, Institute of Clinical Research and Pharmaceutical Expertise, 115088 Moscow, Russia; LelishentsevAA@gmail.com

**Keywords:** *S. baicalensis*, *S. lateriflora*, *S. przewalskii*, S. pycnoclada, hairy roots, HPLC-MS/MS, baicalin, baicalein, wogonin, wogonoside

## Abstract

We measured and studied the growth parameters and the qualitative and quantitative composition of the flavones of hairy roots of the *Scutellaria* genus: *S. lateriflora*, *S. przewalskii* and *S. pycnoclada*. Hairy roots were obtained using wild-type *Agrobacterium rhizogenes* A4 by co-cultivation of explants (cotyledons) in a suspension of *Agrobacterium*. The presence of the *rol*-genes was confirmed by PCR analysis. The hairy roots of the most studied plant from the *Scutellaria* genus, *S. baicalensis,* were obtained earlier and used as a reference sample. HPLC-MS showed the predominance of four main flavones (baicalin, baicalein, wogonin and wogonoside) in the methanol extracts of the studied hairy roots. In addition to the four main flavones, the other substances which are typical to the aerial part of plants were found in all the extracts: apigenin, apigetrin, scutellarin and chrysin-7-*O*-β-d-glucuronide. According to the total content of flavones, the hairy roots of the studied skullcaps form the following series: *S. przewalskii* (33 mg/g dry weight) > *S. baicalensis* (17.04 mg/g dry weight) > *S. pycnoclada* (12.9 mg/g dry weight) > *S. lateriflora* (4.57 mg/g dry weight). Therefore, the most promising producer of anti-coronavirus flavones is *S. przewalskii.*

## 1. Introduction

The search for, and creation of, new medicines based on raw plant materials has attracted the attention of researchers for a long time. These drugs have a wide spectrum of biological action, which makes it possible to use them for the prevention and treatment of various diseases, including coronaviral ones [1,2,3,4]. The special interest should be attracted to the plants of *Lamiaceae* genus, as they are rich in phenolic compounds [5,6,7]. The properties of flavone baicalin as a component of anti-coronavirus drugs are currently being actively studied [8,9]. It is already known that baicalin significantly reduces the oxidative damage to cells, which is induced by the Ang II receptor, and baicalin activates the ACE2-Ang-(1–7) Mas pathway [4]. Baicalin can protect endothelial cells from oxidative stress and Ang II dysfunction by regulation of the PI3K/AKT/eNOS pathway and activation of the ACE2-Ang-(1–7) Mas pathway [10]. The natural producers of baicalin are the members of the genus *Scutellaria*, where it is synthesised in the underground part [11]. In addition to baicalin, plants of the genus *Scutellaria* produce other valuable flavones with proven anti-coronavirus activity—baicalein, wogonin and wogonoside. It has been previously shown that baicalein also inhibited the replication of the SARS-CoV-2 virus and reduced the level of IL- and TNF-a in blood serum [12]. Wogonin and wogonoside are widely known as antitumor agents, but wogonin has also been shown to have an antiviral effect by binding to the main protease of SARS-CoV-2 [13].

One of the main problems in obtaining the necessary components for the production of anti-coronavirus drugs, however, is the lack of raw plant materials. First of all, this is due to the low abundance and slow growth of some species of medicinal plants, as well as the difficulty of creating the essential conditions for their cultivation outside of their natural habitat [14,15]. The attention of scientists is focused on expanding the use of biotechnological approaches to obtain medicinal plant materials containing the target metabolites [16,17,18]. Biotechnology methods which are used to obtain the secondary metabolites include the cultivation of isolated plant tissues and organs in vitro [19,20]. It should be noted that substances obtained from plant raw materials by biotechnology methods usually do not cause side effects, and contain fewer impurities [2,3,21].

Among the species of the genus *Scutellaria*, the following cultures have now been obtained and studied in vitro: *Scutellaria baicalensis*, *Scutellaria lateriflora*, *Scutellaria barbata*, *Scutellaria columnae*, *Scutellaria alpine*, *Scutellaria brevibracteata* and *Scutellaria bornmuelleri* [22,23,24,25,26,27]. The most well-known species of the genus *Scutellaria*—*S. baicalensis*—has been used in traditional Chinese medicine for a long time, and it is the most studied [11]. Comparative experiments have shown that the synthesis of pharmacologically valuable flavones was more effective in the hairy roots culture.

Hairy roots are differentiated cultures of roots obtained through the transformation of a plant with the soil Gram-negative bacterium *Agrobacterium rhizogenes*. The T-DNA of the Ri-plasmid of the *A. rhizogenes* was inserted into plants as a result of this process. Insertion and deletion mutagenesis demonstrated that the development of hairy roots requires the presence of four *rol*-genes (from the root locus): *rol*A, *rol*B, *rol*C and *rol*D [28]. The advantages of hairy roots cultures are the stability of the synthesis of secondary compounds, a high growth rate and cultivation on nutrient media without the addition of hormones [29,30].

Poorly studied species of the genus *Scutellaria,* especially the rare endemic species, are of great interest from the point of view of isolating pharmacologically valuable substances. To identify the most promising producer of biologically active compounds, which could play a key role in the development of drugs for coronaviral infections, in our work, we produced and studied the hairy roots of two species endemic to Uzbekistan—*S. przewalskii* and *S. pycnoclada*. For this purpose, we also compared the qualitative and quantitative composition of their flavonoids with that of the most studied representatives—*S. baicalensis* and *S. lateriflora.*

## 2. Results and Discussion

The first works in obtaining hairy roots of species of the genus *Scutellaria* date back to the end of the twentieth and beginning of the twenty-first century [31,32]. The hairy roots of *S. baicalensis* were the first to be studied. The culture obtained in 2001 by Kuzovkina [33] and deposited in the collection of isolated roots of the Timiryazev Institute of Plant Physiology of the Russian Academy of Sciences was used in our work. There was also research in obtaining similar cultures of the other species of this genus, and there are many studies on *S. lateriflora* and *S. barbata* [34,35,36]. Unfortunately, some endemic species of this genus, which have a narrow habitat and are on the verge of extinction, have not been introduced into in vitro culture yet, including *S. przewalskii* and *S. pycnoclada*.

**Figure 1:**
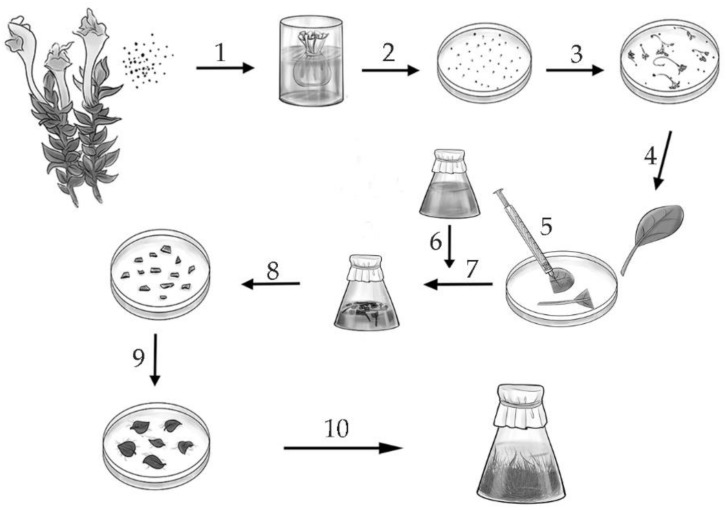
Scheme for obtaining the hairy roots of plants of the genus *Scutellaria* by transformation with *Agrobacterium rhizogenes*. 1—seeds, 2—seed sterilization, 3, 4—seed germination on a solid nutrient medium, 5—damaging explants with an insulin syringe, 6—preparation of a daily culture of *A. rhizogenes*, 7—joint incubation of explants (cotyledons) with *A. rhizogenes* for 12 h, 8—transfer of explants on a solid medium with cefotaxime, 9—formation of hairy roots, 10—growth of hairy roots in a liquid nutrient medium.

The hairy roots of *S. lateriflora*, *S. przewalskii* and *S. pycnoclada* were obtained for this study. The important feature of the transformation method used in this study is the prolonged co-cultivation of explants in an agrobacterial suspension, which, as has been shown previously [37], allowed a higher percentage of transformed explants to be obtained (Figure 1).

When obtaining hairy roots, the percentage of successful transformation for *S. pycnoclada* was 56%, for *S. lateriflora* it was 78% and for *S. przewalskii*, 62%. The *A. rhizogenes* transformation method that we used was quite effective for obtaining hairy roots from a limited amount of starting plant material.

The obtained hairy roots of *S. lateriflora*, *S. przewalskii* and *S. pycnoclada* were tested for the presence of the *rol*-genes by PCR. Only the lines of hairy roots where the presence of all *rol*-genes was confirmed were used for the further studies (Supplementary Figure S1).

As it was shown previously, the *S. baicalensis* hairy roots cultivated on agar and in liquid nutrient media differed significantly from each other in the growth and content of the main flavones [38]. Therefore, in this study, we also compared these indicators in hairy roots growing on media of different density. The results are shown in Table 1.

**Figure 2:**
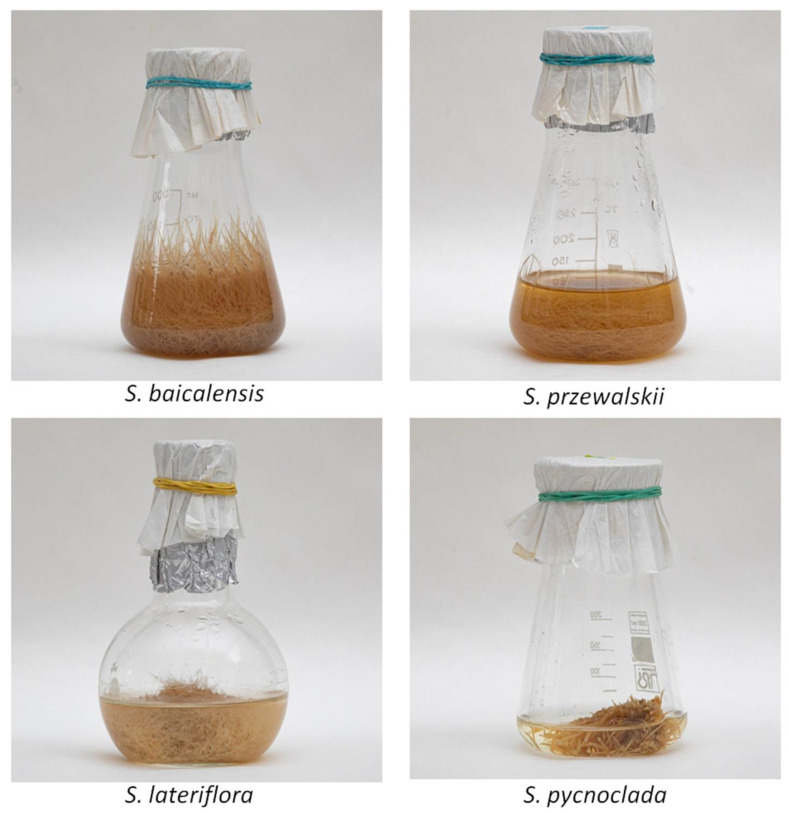
Growth of the hairy roots in a liquid medium.

The growth index was higher when culturing hairy roots on a liquid nutrient medium than on a solid medium (Figure 2).

The hairy roots of *S. baicalensis* cultivated in a liquid nutrient medium had the highest growth index of all the cultures. Interestingly, in the fast-growing hairy roots of *S. baicalensis* and *S. przewalskii*, the growth in liquid and on solid media differed by about nine times, while in slowly growing cultures—*S. lateriflora* and *S. pycnoclada*—this indicator did not differ significantly. The noted differences may be associated with the specific features of the plants, as well as with the location of the insertion of T-DNA into the plant genome. The weaker growth of plant tissues on agar media can be explained by the lower availability of the substrate than during their cultivation in liquid media.

A qualitative analysis of methanol extracts by HPLC-MS/MS from the obtained root cultures of *S. pycnoclada*, *S. przewalskii* and *S. lateriflora* revealed the presence of flavonoid profile of plants of this genus. A total of 19 flavonoids were identified (Table 2), including those specific to the aboveground part of the plants—viscudulin III 6-*O*-β-d-glucoside, apigenin, apigetrin (apigenin-7-*O*-β-d-glucopuranoside), isocarthamidin-7-*O*-β-d-glucuronide, carthamidin-7-*O*-β-d-glucuronide, scutellarin and naringenin. As shown recently, the synthesis of flavones of the aboveground and underground parts in *S. baicalensis* is separated [11]. The detection of flavones such as naringenin, apigenin, apigetrin and scutellarin in the hairy roots culture, which are specific to the aerial part of plants, indicates first of all the activity of the FNSII-1 (flavones-synthase) enzyme. It controls the synthesis of these flavones in the obtained cultures, which is not typical for roots. The roots contain another isoform, FNSII-2 [11]. However, it should be noted that flavones of the aboveground part were present in small or trace amounts in the studied hairy roots.

**Figure 3:**
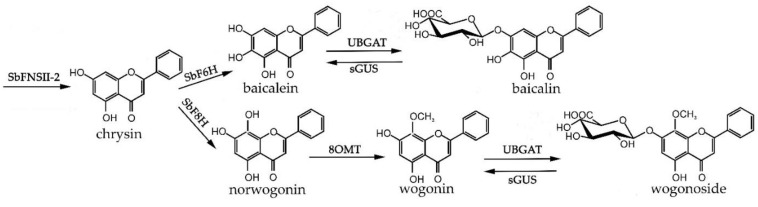
The pathways for synthesis of root-specific flavones (flavones-synthase II (SbFNSII-2) [8], flavones-6-hydroxylase (SbF6H) [39], flavones-8-hydroxylase (SbF8H) [39], 8-*O*-methyltransferase (8OMT) [40], baicalein 7-*O*-glucuronosyltransferase (UBGAT) [41], sGUS—baicalinase, β-glucuronidase [42]).

The studied hairy roots contained significant amounts of root-specific flavones—glucuronides—baicalin and wogonoside, and their aglycones—baicalein and wogonin (Figure 3). Chrysin, a precursor of root-specific flavones, was also present in much smaller amounts.

**Figure 4:**
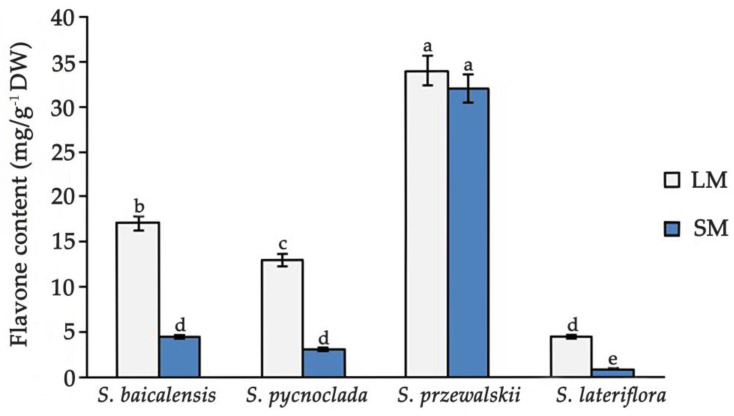
Comparison of the flavones in hairy roots of species of the genus *Scutellaria* (LM—in liquid medium, SM—on a solid medium). Values are presented as the means ± SD. Different letters indicate a significant difference between the means (one-way ANOVA, *p* < 0.05).

According to the content of flavones, plant species form a series on both solid and liquid media: *S. przewalskii* > *S. baicalensis* > *S. pycnoclada* > *S. lateriflora* (Figure 4).

The comparison of the total content of flavones in the hairy roots of all studied species during culture on a solid and in liquid media showed their higher content in a liquid media. According to the ratio of the total content of flavones in a liquid and on solid nutrient medium, hairy roots cultures formed the series: *S. lateriflora* (5.08) > *S. pycnoclada* (4.2) > *S. baicalensis* (3.8) > *S. przewalskii* (1.06). Interestingly, the content of flavones in *S. przewalskii* in liquid and on solid media did not differ. This makes the culture of *S. przewalskii* unique, as it is able to synthesize similar amounts of flavones regardless of the medium density. The content of flavones in the hairy roots of *S. przewalskii* was also much higher than in all the other cultures. Even in the hairy roots of *S. baicalensis*, which have a fairly high synthesizing ability, the content of flavones was almost half as much as in *S. przewalskii*. It is interesting that for the slow-growing hairy roots cultures, *S. lateriflora* and *S. pycnoclada*, the difference in the ratio and content of flavones on different media was higher than in the fast-growing cultures of *S. baicalensis* and *S. przewalskii*. A significant content of flavones thus did not slow down the growth of cultures, since the maximum amounts of flavones were observed in fast-growing cultures.

**Figure 5:**
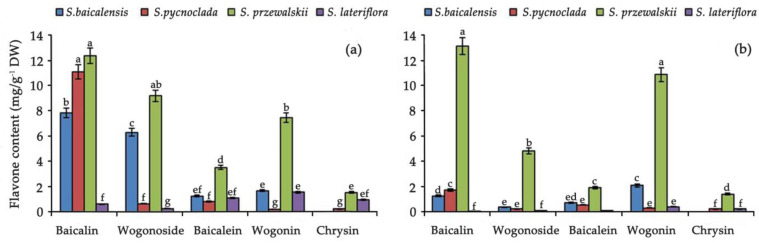
Comparison of the flavones content in the hairy roots of species of the genus *Scutellaria*, (**a**) in liquid medium, (**b**) on solid medium. Values are presented as the means ± SD. Different letters indicate a significant difference between the means (one-way ANOVA, *p* < 0.05).

The highest content of baicalin, which has an anti-coronaviral effect, was found in the hairy roots of *S. pycnoclada* grown on liquid medium, and in the hairy roots of *S. przewalskii* grown on both liquid and solid medium (Figure 5).

As other flavones are present in small amounts, *S. pycnoclada* can be considered a “monoculture” for the synthesis of baicalin (Supplementary Figure S2). Since the growth of *S. pycnoclada* hairy roots during the cultivation cycle is rather low, this culture, however, cannot be used as a promising producer. The content of baicalin in the hairy roots of the most famous representative of the genus *Scutellaria*, *S. baicalensis*, was lower than in the hairy roots of *S. pycnoclada* and *S. przewalskii*. The root culture of *S. lateriflora* had the lowest baicalin content and the lowest growth rate. It should be noted that other works studying the content of individual flavones in the hairy roots of *S. baicalensis* and *S. lateriflora* also demonstrated their lower amounts in *S. lateriflora* [43]. In our study, however, the difference in flavone content between these two hairy roots cultures is much greater.

Interestingly, along with baicalin, the hairy roots of *S. baicalensis* and *S. przewalskii* produced large amounts of another glucuronide, monomethylated wogonoside, which is not dominant in the roots of intact plants. It should be noted that in hairy roots of *S. przewalskii*, the total content of monomethylated flavones (wogonoside and its aglycone wogonin) is at the level of the content of unmethylated flavones (baicalin and its aglycone baicalein). It is also interesting that the content of methylated flavones exceeded the content of unmethylated ones in the hairy roots of *S. lateriflora*. Previously, a similar result was obtained in a study of the hairy roots of *S. baicalensis*, which were also obtained in our laboratory [44,45]. It was suggested that a significant content of methylated flavones is a feature of the hairy roots of this skullcap species, however, the presence of wogonoside and wogonin in *S. przewalskii* and *S. baicalensis* indicated possible changes associated with the insertion of agrobacterial genes during the transformation, which is interesting and requires further study. It is also interesting that baicalin predominated in the hairy roots of *S. pycnoclada*.

Despite the fact that the main attention of researchers is currently focused on baicalin, as a substance with anti-coronaviral properties, there are works that have shown the presence of such antiviral properties for other flavones specific to plants of the genus *Scutellaria* [46]. This allows us to suggest that the cultures containing high concentrations of the entire complex of specific flavones which we studied may show higher efficiency against coronaviruses. Further research is needed to confirm this.

## 3. Materials and Methods

### 3.1. Obtaining the Hairy Roots of S. pycnoclada, S. lateriflora and S. przewalskii, and Determining the Growth Index

Seeds were used to obtain in vitro cultures of *S. pycnoclada*, *S. lateriflora* and *S. przewalskii*. The seeds were sterilized with a 0.1% diocide solution (composition: Ethylmercuric chloride, cetylpyridinium chloride) for 10 min to obtain an aseptic material. Then, they were repeatedly washed with sterile distilled water and transferred for germination in Petri dishes with Murashige-Skoog (MS) medium [47]. The wild strain of *A. rhizogenes* A4 was used to obtain the hairy roots culture. The *A. rhizogenes* suspension culture was grown in YEB liquid medium [48] for 24 h at 23 °C on a circular shaker (amplitude 5–10 cm, rotation speed 90 rpm) before the transformation of the plant material. The hairy roots of *S. pycnoclada*, *S. lateriflora* and *S. przewalskii* were obtained as described previously [37]. The explants (cotyledons and hypocotyls) were incubated in *Agrobacterium* suspension for 12 h, then they were transplanted into fresh B_5_ medium with the addition of 500 mg/L of cefotaxime (Claforan, Great Britain). The explants were transplanted every three days until the complete elimination of the *Agrobacterium*. The appearance of primary roots was observed 14–28 days after transformation. They were separated and transplanted onto agar B_5_ medium with 250 mg/L cefotaxime. The obtained roots were placed in liquid B_5_ medium (the ratio of the volume of the flask and the medium was 100:20) with 250 mg/L of cefotaxime after two passages on the MS medium with agar addition (Figure 1). After four weeks of cultivation, hairy roots were transferred to the medium without antibiotics. The cultivation cycle was four weeks. The plant material was cultured in the dark at 23 °C in a shaker at a rate of 90 rpm.

We used the growth index (I) to characterize the growth of hairy roots, which was calculated using the following equation:

I = (X_max_ − X_0_)/X_0_,

where X_max_ is the highest raw weight level achieved by the culture (g), and X_0_ is the starting raw weight of the culture (g). The initial weight of the roots and calluses was 0.5 g. Graphs and tables show the average arithmetic values of growth parameters for three to four biological repetitions of each variant.

### 3.2. DNA Isolation and PCR Analysis

The total genomic DNA from 100 mg of fresh plant material was extracted using a modified CTAB method [49], where the samples are supplemented with polyvinyl pyrrolidone to avoid binding polyphenols to DNA chains, and subsequently purified twice with chloroform. The DNA concentration in the samples was estimated using a ND-1000 spectrophotometer (NanoDrop Technologies, Wilmington, DE, USA).

Transformation was confirmed by PCR detection T-DNA using primers spanning the *rol*A, *rol*B, *rol*C and *rol*D genes (Table 3). The PCR reactions were carried out in a final volume of 25 µL, containing 40 ng of template DNA, 0.2 mM of each dNTP and 2.5 µL of 10X PCR Buffer (Qiagen) containing 1.5 mM (final concentration) of MgCl_2_, 2.5 U Taq DNA polymerase (Qiagen) and 0.3 µL of each primer. The PCR amplification was performed using the programmed thermocycler MS2 (DNK-Tekhnologiya, Moscow, Russia). The thermal cycling program consisted of initial denaturation for 2 min at 94 °C; 5 cycles: denaturation for 20 s at 94 °C, annealing for 10 s at 60–62 °C (Table 1), elongation for 10 s at 72 °C; 40 cycles: denaturation for 5 s at 94 °C, annealing for 5 s at 60–62 °C, elongation for 5 s at 72 °C; final elongation for 2 min at 72 °C. The amplified products were separated by electrophoresis in a 2% agarose gel with 0.01% ethidium bromide in 1X TBE buffer and visualized by fluorescence under UV light.

### 3.3. Preparation of the Extracts and Determination of Secondary Metabolite Contents

The extraction of freeze-dried biomass from samples was performed with methanol (1:100 biomass: extractant ratio) in an FS14H ultrasonic bath (Fisher Scientific, Waltham, MA, USA) for 180 min, then 1 mL of the extract was taken and centrifuged for 10 min at 8000 rpm. The volume of 0.85 mL of supernatant was taken and used for high-pressure liquid chromatography (HPLC).

### 3.4. HPLC

The separation of flavones was carried out using the Agilent 1260 (Agilent 1260 VWD, Agilent Technologies, Waldbronn, Germany) with variable wavelength (VWD 1260) in a gradient elution mode. The chromatographic column was an Agilent Zorbax C-18 (150 × 4.6 mm, 3.5 µm). The column oven was set at 30 °C. The mobile phase consisted of 0.1% trifluoroacetic acid in ultrapure water (solvent A) and acetonitrile (solvent B). The mode with gradient and isocratic constituents was used for the separation: 0 min—42% A, 8 min—42% A, 26 min—34% A, 28 min—42% A, 33 min—42% A. The flow rate was 0.6 mL/min. The volume injected was 5 µL. The detection was carried out at λ = 275 nm. The flavonoid peaks were identified by comparing their UV spectra and retention times with the corresponding parameter of the chromatographically pure baicalin and baicalein made by AppliChem (Darmstadt, Germany), wogonoside and wogonin made by Sigma, chrysin standards made by Roth. The chromatograms were processed using the “ChemStation” software. The contents of the studied flavones were determined using the calibration curves plotted in the concentration ranges 1–100 mkg/mL (Table 4).

### 3.5. HPLC-MS/MS

A mass spectrometric study was carried out with the obtained methanol extracts. The samples were diluted with methanol (10 times). The mass spectrometric analysis was performed on a tandem mass spectrometer by Thermo TSQ Endura (Thermo Fisher Scientific, San Jose, USA). The analysis was carried out in the full-scan (for detecting peaks with maximum intensity) and MRM (multiple reaction monitoring) modes (for analyzing fragment ions, and for identifying compounds). The test substances were determined by electrospray ionization in the positive region. A number of sources were used to confirm the information received and to compile the list of analyzed compounds [50,51,52,53,54].

### 3.6. Statistical Analysis

Statistical analysis was performed using a one-way ANOVA test. All the experiments were performed in triplicate with at least three independent runs. The data are presented in the tables and in the figures as means ± SD. Different symbols show significantly different values. Mean values were considered significantly different at *p* < 0.05.

## 4. Conclusions

The hairy roots of *S. pycnoclada* and *S. przewalskii* were obtained and composition of their flavonoids was studied. The qualitative composition of their methanol extracts was characteristic of plant species of the genus *Scutellaria*. The quantitative study of the content of the main flavones in obtained cultures demonstrated that in the hairy roots of *S. pycnoclada,* the predominant flavone was baicalin. Therefore, this culture could be considered a mono-flavone culture. The low growth index, however, does not make it possible to classify this culture as an effective producer of baicalin, which has an anti-coronavirus effect. The highest content of baicalin and other flavones was observed in the hairy roots culture of *S. przewalskii*. It also has a unique feature—the same level of synthesis of main flavones on agar and liquid nutrient media. The high synthesis level of target metabolites combined with the high growth index makes the hairy roots culture of *S. przewalskii* the most promising producer of antiviral flavones.

## Figures and Tables

**Table 1:** Growth index of the hairy roots of *S. baicalensis, S. lateriflora, S. przewalskii* and *S. pycnoclada* at the end of the cultivation cycle on agar and liquid nutrient medium.

Hairy Roots Growing Medium	*S. baicalensis*	*S. lateriflora*	*S. przewalskii*	*S. pycnoclada*
Liquid medium	43 ± 3.8 ^a^	6.4 ± 2.7 ^c^	32 ± 1.6 ^b^	4.7 ± 0.5 ^c^
Solid medium	5.9 ± 0.7 ^c^	5.1 ± 1.2 ^c^	4.8 ± 0.8 ^c^	1.9 ± 0.1 ^d^

Values are presented as the means ± SD and different letters represent significant differences at *p* ≤ 0.05 according to one-way ANOVA.

**Table 2:** Identification of flavones in hairy roots *S. baicalensis*, *S. lateriflora*, *S. przewalskii* and *S.pycnoclada* by HPLC-MS/MS.

№	Compounds	Molecular Mass	MRM Transition, *m*/*z*
1	baicalin	446	447.1 → 271.1
2	wogonoside	460	461.1 → 285.1
3	baicalein	270	271.1 → 123.1
4	wogonin	284	285.1 → 270.1
5	chrysin	254	255.1 → 153.1
6 *	chrysin-6-*C*-β-d-glucoside	416	417.1 → 281.1
7 *	chrysin-8-*C*-β-d-glucoside		
8	tenaxin	344	345.1 → 330.1
9	viscudulin III 6-*O*-β-d-glucoside	508	509.1 → 347.1
10	apigenin	270	271.1 → 153.1
11	apigetrin (apigenin-7-*O*-β-d-glucopuranoside)	432	433.1 → 271.1
12 *	isocarthamidin-7-*O*-β-d-glucuronide	464	465.1 → 289.1
13 *	carthamidin-7-*O*-β-d-glucuronide		
14	scutellarin	462	463.1 → 287.1
15	chrysin-7-*O*-β-d-glucuronide	430	431.1 → 255.1
16	baicalein-7-*O*-β-d-glucoside	432	433.1 → 415.1
17	naringenin	272	273.1 → 153.1
18	oroxylin A	284	285.1 → 267.1
19	scullcapflavone II	374	375.1 → 345.1

* Structural isomers. In the hairy roots of *S. baicalensis*, 1–16 were found; *S. pycnoclada*, 1–16; *S. przewalskii*, 1–5, 7–14, 16–19; *S. lateriflora*, 1–7, 9–16, 18.

**Table 3:** Nucleotide sequence of primers used for detection of *rol*-genes in hairy root cultures.

Genes	Nucleotide Sequence (nt)	Tm (°C)
*rol*A	5′-ggaaatccgcaatcaac-3′	62
	5′-tttgcacgcctaacaag-3′	
*rol*B	5′-GCTCTTGCAGTGCTAGATTT-3′	60
	5′-GAAGGTGCAAGCTACCTCTC-3′	
*rol*C	5′-CTCCTGACATCAAACTCGTC-3′	60
	5′-TGCTTCGAGTTATGGGTACA-3′	
*rol*D	5′-CATCTGCAACTGAGCGTGTG-3′	62
	5′-TGTCTGATAGGGAGGAACGA-3′	

**Table 4:** Calibration curve data.

Flavone	Calibration Range (µg/mL)	R^2^
baicalin	1–100	0.9997
wogonoside	1–100	0.9986
baicalein	1–100	0.9988
wogonin	1–100	0.9998
chrysin	1–50	0.9993

## Data Availability

The data presented in this study are available upon request from the corresponding author.

## Supplementary Materials

The following are available online. Figure S1: PCR amplification of *rol*A, *rol*B, *rol*C and *rol*D genes in the hairy roots of species of the genus *Scutellaria*. Figure S2: HPLC chromatogram of the hairy roots methanol extract.

## Sample Availability

Samples of the compounds are available from the authors.
