# Supplementary material for: Hairy Roots of Scutellaria spp. (Lamiaceae) as Promising Producers of Antiviral Flavones
Source: Molecules. 2021 Jun 27;26(13):3927. doi: 10.3390/molecules26133927 (PMC8271535; doi:10.3390/molecules26133927)
Supplement: Supplementary file 1 [file molecules-26-03927-s001.zip › molecules-1262209-supplementary.pdf]

Supplementary data

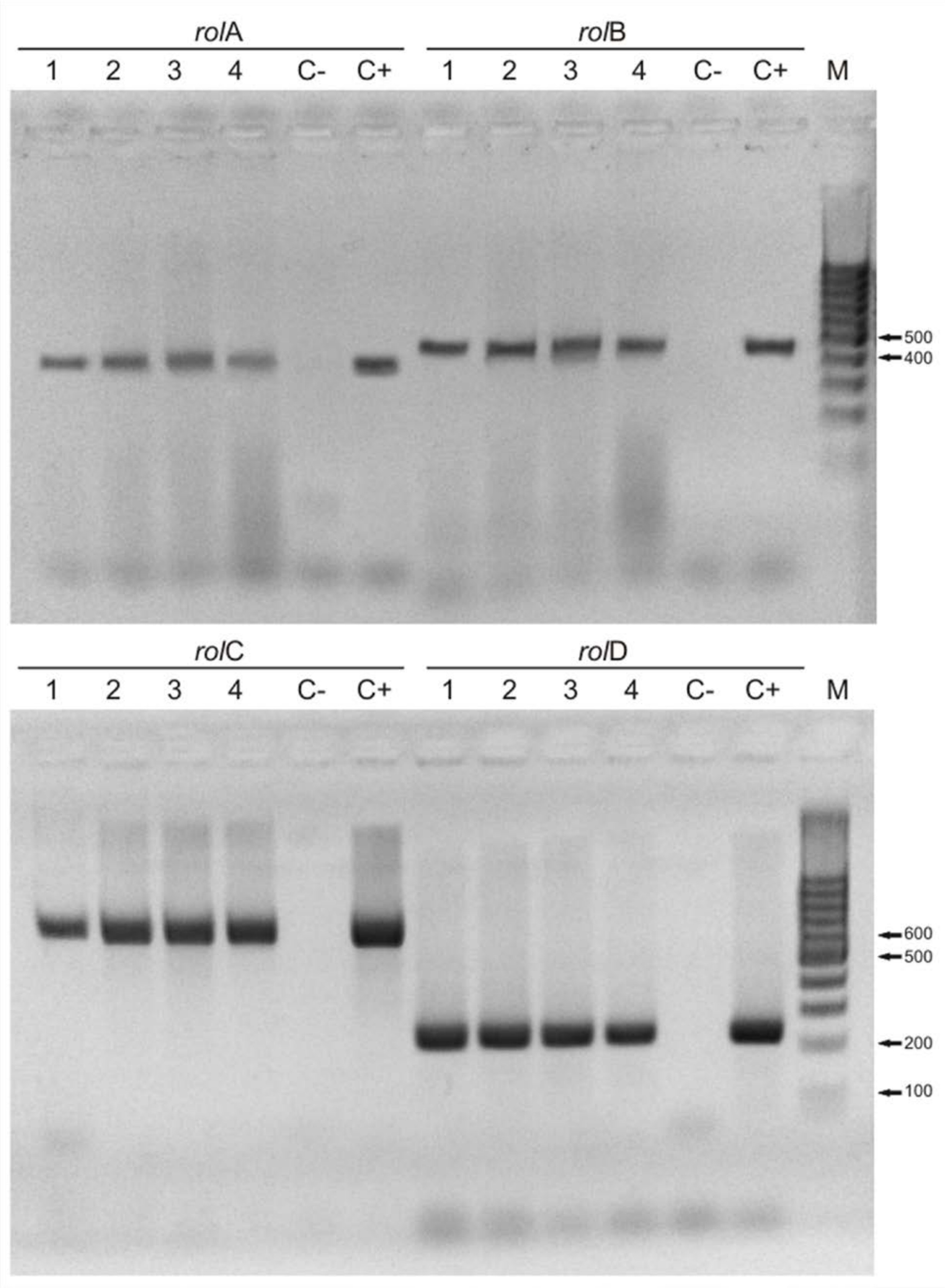

Supplementary Figure S1. PCR amplification of *rol*A, *rol*B, *rol*C, *rol*D genes in the hairy roots of species of the genus *Scutellaria.* Lane 1: DNA from the hairy roots of *S. baicalensis*; lane 2: *S. lateriflora*; lane 3: *S. przewalskii*; lane 4: *S. pycnoclada*; M: molecular markers (100 bp ladder); C-: negative control, C+: *A. rhizogenes* strain A4 (positive control).

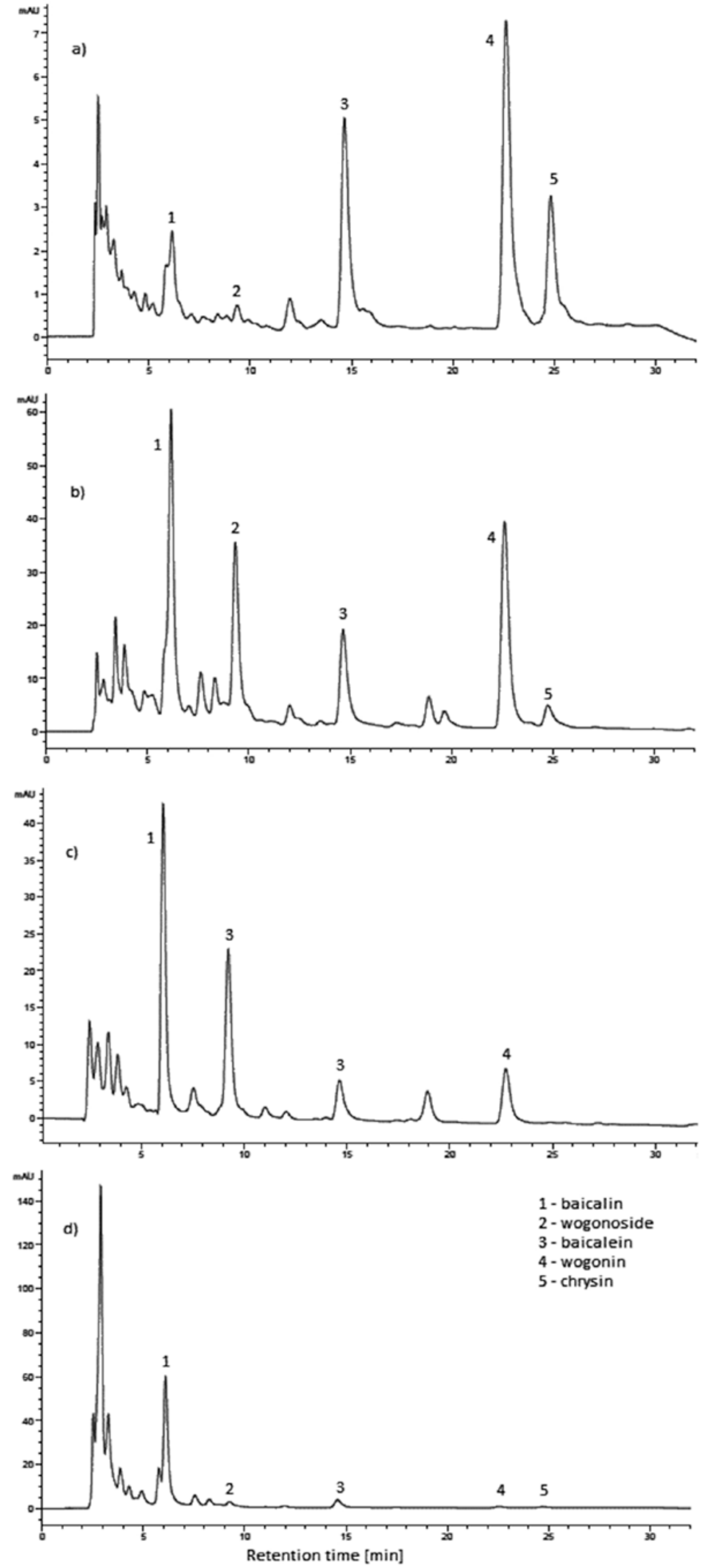

Supplementary Figure S2. HPLC chromatogram of the hairy roots methanol extract: a) *S. lateriflora;* b) *S. przewalskii;* c) *S. baicalensis;* d) *S. pycnoclada.*
